# Pharmacokinetics-Driven Evaluation of the Antioxidant Activity of Curcuminoids and Their Major Reduced Metabolites—A Medicinal Chemistry Approach

**DOI:** 10.3390/molecules26123542

**Published:** 2021-06-10

**Authors:** Gábor Girst, Sándor B. Ötvös, Ferenc Fülöp, György T. Balogh, Attila Hunyadi

**Affiliations:** 1 Institute of Pharmacognosy, Interdisciplinary Centre of Excellence, University of Szeged, H-6720 Szeged, Hungary; girst.gabor@pharmacognosy.hu; 2 Institute of Pharmaceutical Chemistry, University of Szeged, H-6720 Szeged, Hungary; sandor.oetvoes@uni-graz.at (S.B.Ö.); fulop.ferenc@szte.hu (F.F.); 3 Institute of Chemistry, University of Graz, NAWI Graz, Heinrichstrasse 28, A-8010 Graz, Austria; 4 Department of Chemical and Environmental Process Engineering, Budapest University of Technology and Economics, H-1111 Budapest, Hungary; 5 Department of Pharmacodynamics and Biopharmacy, University of Szeged, H-6720 Szeged, Hungary; 6 Interdisciplinary Centre of Natural Products, University of Szeged, H-6720 Szeged, Hungary

**Keywords:** curcumin metabolite, continuous-flow hydrogenation, physicochemical characterization, gastrointestinal and blood-brain barrier penetration, pharmacokinetics, metabolism

## Abstract

Curcuminoids are the main bioactive components of the well-known Asian spice and traditional medicine turmeric. Curcuminoids have poor chemical stability and bioavailability; in vivo they are rapidly metabolized to a set of bioreduced derivatives and/or glucuronide and sulfate conjugates. The reduced curcuminoid metabolites were also reported to exert various bioactivities in vitro and in vivo. In this work, we aimed to perform a comparative evaluation of curcuminoids and their hydrogenated metabolites from a medicinal chemistry point of view, by determining a set of key pharmacokinetic parameters and evaluating antioxidant potential in relation to such properties.Reduced metabolites were prepared from curcumin and demethoxycurcumin through continuous-flow hydrogenation. As selected pharmacokinetic parameters, kinetic solubility, chemical stability, metabolic stability in human liver microsomes, and parallel artificial membrane permeability assay (PAMPA)-based gastrointestinal and blood-brain barrier permeability were determined. Experimentally determined logP for hydrocurcumins in octanol-water and toluene-water systems provided valuable data on the tendency for intramolecular hydrogen bonding by these compounds. Drug likeness of the compounds were further evaluated by a in silico calculations. Antioxidant properties in diphenyl-2-picrylhydrazyl (DPPH) radical scavenging and oxygen radical absorbance capacity (ORAC) assays were comparatively evaluated through the determination of ligand lipophilic efficiency (LLE). Our results showed dramatically increased water solubility and chemical stability for the reduced metabolites as compared to their corresponding parent compound. Hexahydrocurcumin was found the best candidate for drug development based on a complex pharmacokinetical comparison and high LLE values for its antioxidant properties. Development of tetrahydrocurcumin and tetrahydro-demethoxycurcumin would be limited by their very poor metabolic stability, therefore such an effort would rely on formulations bypassing first-pass metabolism.

## 1. Introduction

Curcumin, and its analogs (demethoxycurcumin: DMC, and bisdemethoxycurcumin) can be found in many species from the ginger family (Zingiberaceae) such as in turmeric (*Curcuma longa*), wild turmeric (*Curcuma aromatica*) and Javanese turmeric (*Curcuma xanthorrhiza*). Curcumin is the main bioactive compound of the widely spread “golden spice”, turmeric, which has been used in traditional Chinese and Ayurvedic medicine for centuries [1]. Natural product research in general has been experiencing a new renaissance recently, which is no wonder considering the high proportion of natural product-originated or -inspired compounds among approved drugs [2]. Curcumin is one of the most studied natural products, with well over 2000 hits for publications on PubMed only for 2020. It was reported to exert a plethora of promising bioactivities in vitro and in vivo, including anti-inflammatory [3], antimetastatic [4,5], antiangiogenic [6], antitumor and anti-mutagenic [7], chemo-preventive [8], neuroprotective [9], etc. Its popularity is also shown by the several currently ongoing clinical trials on curcumin, even though it is now recognized as a pan-assay interference-substance (PAINS). This is because of its promiscuous action in a wide variety of in vitro bioassays, giving many false “hits” through non-specific binding to proteins due to its reactivity or interfering with readouts due to its spectroscopic properties [10,11]. Because of this, curcumin itself is not a drug-like compound.

Another major issue about the use of curcumin as a potential therapeutic is that it has poor pharmacokinetic properties, low bioavailability and chemical stability, and a rapid metabolism. It was reported that its human consumption is safe in up to 12 g/day amounts, but its absorption is really poor [12]. Because of curcumin’s reactive structure the absorbed amount has a high potential to be metabolized, which was extensively studied in human liver microsomes. In phase I metabolism, the double bonds of curcumin are saturated primarily by the alcohol dehydrogenase route [13]. It is important to stress that a partial or full saturation of the alkyl chain of curcumin eliminates its PAINS properties, therefore these reduced metabolites do not anymore fall into this problematic category of compounds. In Phase II processes the phenolic moiety of curcumin and its reduced derivatives are rapidly conjugated to glucuronides and sulphates.

Some of the main metabolites of curcumin are tetrahydrocurcumin (4HC), hexahydrocurcumin (6HC) and octahydrocurcumin (8HC). These compounds are formed by reductive transformation in human Phase I metabolic route, however that is not the only biotransformation pathway as they can be identified. The curcuminoids 4HC and 8HC have been found in *Curcuma longa*, in the rhizomes and in the leaves, respectively, and 6HC naturally occurs in the rhizomes of *Zingiber officinale* [14,15,16]. Synthetically the easiest way to produce them is by the catalytic hydrogenation of curcumin. In traditional batch reaction, using Palladium on activated carbon as catalyst, all three can be synthesized, and various yields had been reported, ranging from 14 to 68% for 4HC, 9 to 20% for 6HC and 0 to 15% for 8HC [17,18,19]. Using autoclaves at high pressure 8HC can be produced with as high as 92% yield [20], and it is also synthesizable from 4HC, with 74% yield using sodium tetrahydroborate as reagent [21].

Continuous flow hydrogenation typically has many advantages compared to the traditional batch hydrogenation in terms of safety, optimization speed of reaction conditions, reaction efficiency/space-time yields (STY), and residence time/reaction rates. The necessary explosive hydrogen gas is generated in situ and elevated pressure can also be easily provided. Also, the reaction conditions are more controllable, and in most cases this method shows better selectivity [22].

The continuous flow (CF) hydrogenation of curcumin is not quite explored. So far only two articles can be found where the reduction took place under CF conditions. Wagner et al. [23] in 2013 developed an educational laboratory experiment where students use the H-cube continuous flow hydrogenation reactor to prepare 4HC from curcumin. It is a visible reaction as the bright yellow solution of curcumin turns colorless upon saturation. In the supplementary info, they state that the catalytic reaction in this way gives 92–100% yield compared to the 60% achieved in a batch reaction. They used 10% Pd/C as catalyst, and ethanol—ethyl acetate (1:1, *v*/*v*) as solvent at 60 °C with a flow rate of 1 mL/min. Batie et al. [24] in 2013 also used an H-cube to reduce the double bonds of a halogenated-curcumin derivative to tetrahydrohalogenated curcumin with 49% yield. In these articles they did not mention anything about why the described conditions were chosen.

There are several in vitro and in vivo experimental evidences that these hydrocurcumin derivatives exhibit similar or even better bioactivity than curcumin such as antioxidant [25], anti-inflammatory [26], antitumor [27] and cardiovascular protective properties [28]. 4HC and 8HC have better hepatoprotective effect than curcumin against acetaminophen-induced liver injury and possess superior anti-inflammatory effects in vivo through suppression of TAK1-NF-κB pathway [29,30,31].

The antioxidant activity of the three main reduced derivatives (4HC, 6HC, 8HC) have been thoroughly investigated by Somparn et al., and they concluded that hydrogenated curcumins show better DPPH-scavenging activity and are stronger antioxidants against lipid peroxidation and red blood cell hemolysis [25].

As a part of the preclinical evaluation, extensive characterization of the in silico/in vitro physicochemical and ADME properties is an effective tool to decrease a relative high attrition of active compounds in early stage drug discovery. This approach is widely used for primary screening and preclinical candidate selection, in which the main parameters are kinetic solubility, chemical stability, acid/base properties (p*K_a_*), lipophilicity (log*P*/*D*), in vitro gastrointestinal and CNS-specific permeability (P_e_, P_app_) [32] and liver microsomal study focusing on cytochrome P450-mediated metabolic stability (Cl_int_, t_1/2_) [33,34].

In the present work, our aim was to further investigate the possibility of the hydrogenation of curcumin in continuous flow reactor, and to see how changing the basic reaction conditions could influence the selectivity of catalytic reduction. Furthermore, we aimed to get more information about the in silico and in vitro physicochemical and pharmacokinetic characteristics of the reduced curcumin metabolites, and to evaluate their antioxidant potential in relation to those properties.

## 2. Results and Discussions

### 2.1. Continuous-Flow Hydrogenation of Curcumin

**Figure 1:**
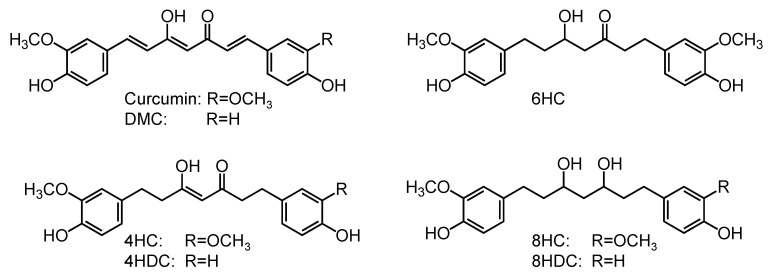
Structures of the curcuminoids and their reduced derivatives studied in this work. DMC: demethoxycurcumin, 4HC: tetrahydrocurcumin, 4HDC: tetrahydro-demethoxycurcumin, 6HC: hexahydrocurcumin, 8HC: octahydrocurcumin, 8HDC: octahydro-demethoxycurcumin.

For the hydrogenation of curcumin, we tried several reaction conditions utilizing the H-cube, including concentration, solvent, pressure, temperature, and flow rate. Yields and space time yields (STY) for the hydrogenated metabolites 4HC, 6HC and 8HC are shown in Table 1 for each tested condition. Structures of the compounds, along with DMC and its reduced derivatives also studied in this work, are shown in Figure 1.

In the case of methanol as solvent, increasing the pressure led to an increased selectivity towards tetrahydrocurcumin (4HC). When using a less polar solvent, toluene, in which the solubility of curcumin is worse, at room temperature 8HC was barely formed, which could be explained by the shifted keto-enol tautomer equilibrium. Increasing the residence time by lowering the flow rate does not improve the selectivity towards the more saturated compounds. Increasing the concentration of the substrate seems to change the selectivity towards 4HC.

With these results we have a better understanding about how the basic parameters influence the selectivity of the hydrogenation of curcumin, using continuous flow hydrogenation reactor. This method might not have the best achievable selectivity, it is a good and safer way to produce hydrogenated curcumin metabolites.

### 2.2. In Silico and Experimental Physicochemical and Pharmacokinetic Characterization

Because curcumin and its reduced metabolites cover a narrow chemical space, a multiparametric characterization of the compounds is of high relevance. This was performed using in silico and experimental ADME-related parameters (Table 2 and Table 3). Comparing the predicted and measured aqueous kinetic solubility data, it was found that the solubility of the hydrocurcumins is much higher than that of curcumin and its demethoxy analog (DMC). Furthermore, both datasets indicate the enhanced solubility of 6HC. The metabolites (4H(D)C—6H(D)C) did not show any chemical degradation in PBS (pH 7.4) medium at 37 °C after 2 h, while relatively short shelf-life was found for curcumin and DMC respectively. This is well in line with previous studies on the chemical instability of curcumin under such conditions; as reported by Wang et al., curcumin suffers a rapid oxidative fragmentation in cell culture medium, which should play a significant role in the pharmacological promiscuity of this compound [35]. Interestingly, human liver microsomes metabolize 4HC and 4HDC even faster than curcumin, showing an order of intrinsic clearance (Cl’_int_) as 4HC ≈ 4 HDC > DMC > curcumin > 6HC >> 8HC > 8HDC. A semi-qualitative investigation of microsomal transformation was also performed by characteristic mass change of curcumin and its analogs using LC-MS data (see Supporting Information, Table S1). Based on the change of *m*/*z* values from parent compounds (M) as a starting material and similarity in retention times, the formation of 6HC as a major metabolite was identified by the reductive metabolism of curcumin (M + 6) and 4HC (M + 2) and also by the oxidative transformation of 8HC (M-2) in the microsomal system. It is also important to note that most of the reductive metabolic routes (M + 2, +4 and +6) were also identified for both curcumin and the hydrocurcumins. This result is also of interest because previous studies connected the formation of reduced metabolites of curcumin to the cytosolic alcohol dehydrogenase [36]. In our case, the formation of reduced metabolites is most likely attributable to the Cytochrome P450 reductase activity from the NADPH-dependent enzyme pool of the microsomal system. Thus, our results also suggest a potential alternative metabolic pathway of curcumin and its reduced derivatives.

Due to the almost complete GI-specific lipid membrane retention (MR), the gastrointestinal permeability of curcumin and DMC could not be determined, while its reduced metabolites followed the 4HC > 4HDC > 6HC > 8HC > 8HDC order from medium to low. A similar situation was identified in the blood-brain barrier-specific lipid permeability assay, i.e., the decreased permeability of curcumin and DMC correlated with their elevated membrane retention. In this case, the BBB permeability order was found as 4HC > 4HDC > DMC > curcumin > 6HC ≈ 8HC ≈ 8HDC (Table 2). A similar order to the experimental data can be identified based on the corresponding predicted values (CNS MPO, logBB, Caco-2 permeability) for the drug absorption property of curcumin and its metabolites. A more detailed overview of lipophilicity (Table 3) and permeability (GI-PAMPA, Table 2) data revealed a slightly confusing relationship between hydrocurcumin derivatives. While the kinetic solubility of these compounds, which also affects their PAMPA-based permeability, is nearly identical, unclear positive correlation can be shown between predicted lipophilicity values and P_e_ data. Considering the structure of hydrocurcumins, a possible explanation for this phenomenon is the existence of intramolecular hydrogen bonds (IMHB), which improves the ADME properties of drugs, especially their absorption as suggested by several studies [37,38,39]. To confirm the presence of intramolecular interactions in these compounds, the partition behavior of hydrocurcumins was also investigated in both octanol—water (logP_oct/w_) and toluene—water (logP_tol/w_) systems. It has been shown that the difference between logP values obtained in two different biphasic systems (∆logP_oct_tol_ = logP_o/w_ − logP_tol/w_) is informative of the solutes IMHB properties [39,40,41]. Based on previous observations, if the increased lipophilicity and the ∆logP_oct_tol_ of a compound is close to zero, this indicates the high propensity to form IHBD. Investigating the lipophilic character of three structurally relevant hydrocurcumins in two biphasic systems (Table 4) increased lipophilicity, and lowest ∆logP_oct_tol_ were identified for 4HC to suggest highest tendency to IMHB in the set of compounds. The experimental logP_oct/w_ values of 4HC, 6HC and 8HC correspond to the order of the predicted logP values (Table 3), and the ∆logP_oct_tol_ data explain the order of GI- and BBB-specific permeability of 4HC > 6HC > 8HC, which is also supported by the same order of probability of IMHB.

Since in the reduced metabolites the connecting chain does not have any double bonds conjugated with the two aromatic moieties, demethoxylation cannot have an influence on the IMHB probability, therefore DMC and its reduced metabolites must follow the same tendency.

Overall, due to its favorable solubility, chemical stability, optimal liver microsomal stability and moderate permeability property, 6HC has the best properties among curcumin and its reduced metabolites from a medicinal chemistry point of view. However, due to the increased GI- and BBB-specific penetration of 4HC and 4HDC, these may also be suitable clinical candidates with a drug delivery strategy that bypasses first-pass metabolism. It is also important to highlight our finding that 6HC were identified as a recurrent metabolite of both curcumin and its reduced analogs.

### 2.3. Antioxidant Activity

The antioxidant activity of curcumin, demethoxycurcumin and their hydrogenated derivatives have been comparatively investigated using multiple models [25]. To evaluate their in vitro free radical scavenging activity in relation to our pharmacokinetic results, we completed our study by measuring the capacity of these compounds to scavenge diphenyl-2-picrylhydrazyl (DPPH) radicals and by determining their oxygen radical absorbance capacity (ORAC); results are compiled in Table 5.

These two methods offer some complementary information due to their reaction mechanisms with small-molecule antioxidants: DPPH may react through single-electron transfer (SET) or hydrogen atom transfer (HAT) mechanism, while the ORAC assay works mainly through HAT [40]. Our results correlate with those published before, i.e., 4HC shows the best activity against DPPH, while the demethoxy derivatives the worst.

Curcumin, DMC and 4HDC showed the best results in the ORAC assay. It is important to mention that such chemical antioxidant assays alone have low in vivo relevance [41]. Nevertheless, they still provide valuable input data to compare the antioxidant potential of related compounds when corrected with a relevant pharmacokinetic property to obtain a related ligand efficiency (LLE) measure that is an approach of major importance in drug discovery [44]. Accordingly, when calculating lipophilic ligand efficiency to both applied free radical scavenging assays, hexahydrocurcumin (6HC) was found to be by far the best lead among the compounds studied, and the only derivative that was superior to both its parent compound, curcumin, and trolox in both assays according to its LLE value.

## 3. Materials and Methods

### 3.1. Synthesis and Chromatographic Purification

Curcumin (≥98% curcuminoid, ≥65% curcumin content) and all other reagents were purchased from Sigma (Merck KGaA, Darmstadt, Germany) and the solvents were obtained from Macron Fine Chemicals (Avantor Performance Materials, Center Valley, PA, USA).

For the purification of curcumin a flash method was selected based on Jayaprakasha et al. [45] using silica (40 g) and diol (30 g) columns in series. For this a CombiFlashfi Rf + Lumen apparatus (TELEDYNE Isco, Lincoln, NE, USA) was utilized that was equipped with ELS and diode array detectors.

Using various conditions curcumin was reduced to tetrahydrocurcumin, hexahydrocurcumin and octahydrocurcumin, utilizing an H-cube continuous flow hydrogenation reactor (ThalesNano Inc., Budapest, Hungary). For the analytic evaluation of the products RP-HPLC analysis was selected, using a gradient of acetonitrile and water (from 40% MeCN to 80% in 30 min) with a Kinetex Biphenyl 250 × 4.6 mm, 5 µm column (Phenomenex Inc., Torrance, CA, USA) with a flow rate of 1 mL/min, using a dual pump (PU-2080) Jasco HPLC instrument (Jasco International Co. Ltd., Hachioji, Tokyo, Japan) with an MD-2010 Plus PDA detector. For the isolation of the products an Armen Spot Prep II integrated HPLC purification system (Gilson, Middleton, WI, USA) with dual-wavelength detection was applied, operating at 220 and 254 nm. Preparative separations were performed on a Kinetex Biphenyl 250 × 21.2 mm, 5 µm column with adequately chosen eluents of acetonitrile–water, and the flow rates were 15 mL/min.

### 3.2. Pharmacokinetic Measurements

Kinetic solubility was tested with 5% (*v*/*v*) DMSO as cosolvent in phosphate buffered saline (PBS, 0.01 M phosphate buffer pH 7.4, 0.138 M NaCl, 0.0027 M KCl, without Ca^2+^, Mg^2+^) at room temperature for 2 h. Chemical stability was tested with 10% (*v*/*v*) DMSO as cosolvent in PBS pH 7.4 at 37 °C for 4 h. Metabolic stability was investigated on human liver microsomes (1 mg/mL), which were preincubated with 25 µM test compounds for 5 min at 37 °C in 0.1 M Tris-HCl buffer, pH 7.4 contained 6.67 mM Na-pyrophosphate, 5.56 mM MgCl_2_, 5.56 mM glucose-6-phosfate, 4.63 U/mL glucose-6-phosphate dehydrogenase. The reaction was initiated by adding preincubated cofactor (37 °C,10 mM NADPH). After 10, 30, and 60 min incubation at 37 °C, the reaction was stopped by adding equal volume of cooled methanol (−20 °C). The samples were centrifuged at 20,000 g for 5 min. The supernatant and all above described test solutions were analyzed by LC-MS/MS. LC-MS analysis was carried out on an Agilent 1200 liquid chromatography system coupled with an 6410 QQQ-MS (Agilent Technologies, Palo Alto, CA, USA). Analysis was on a Cortecs C18 column (150 × 4.6 mm, 2.7 µm, Waters) at 40 °C, with a mobile phase flow rate of 1.45 mL/min. Composition of eluent A was 0.1% (*v*/*v*) trifluoroacetic acid (TFA) in water (pH 1.9). A linear gradient of 0–100% B (acetonitrile (AcN) and water in 95:5 (*v*/*v*)) was applied at a range of 0–17 min, then 100% B at 17–22 min. The injection volume was set at 20 µL and chromatograms were registered at 220 ± 4 nm. The MSD operating parameters in ESI positive ionization mode were as follows: scan ion mode (100–800 *m*/*z*), drying gas temperature 350 °C, nitrogen flow rate 12 L/min, nebulizer pressure 40 psi, quadrupole temperature 100 °C, capillary voltage 4000 V, fragmentor voltage 135 V.

BBB and GI permeability was determined by using tissue-specific parallel artificial membrane permeability assay (GI-PAMPA [46], BBB-PAMPA [47]) models. Briefly, a 96-well acceptor plate and a 96-well filter plate were assembled into a sandwich. The hydrophobic filter material of the 96 well filter plate was coated by the mixture of phosphatidylcholine:cholesterol 2:1 (5 µL, 4 % (*w*/*v*) in dodecane: GI-PAMPA) or by porcine brain lipid (PBL) (5 µL, 2.6 % (*w*/*v*) in dodecane:hexane 25:75 *v*/*v*%: BBB-PAMPA). Subsequently, the acceptor wells at the bottom of the sandwich were filled with 300 µL of 10 mM PBS solution with 5% DMSO adjusted to pH 7.4. The donor wells at the top of the sandwich were hydrated with 150 µL of test compound solution. The test compound solutions were prepared in 100 times dilution. Firstly, a 10 mM stock solution was prepared from the corresponding compound in DMSO, then it was diluted with PBS (pH 6.5: GI-PAMPA or pH 7.4: BBB-PAMPA) to get 5% mixture followed by filtration through a MultiScreen Solubility filter plate. The resulting sandwich was then incubated at 37 °C for 4h. After the incubation, PAMPA sandwich plates were separated and compound concentrations in donor and acceptor solutions were investigated by HPLC-DAD. HPLC analyses were performed using a Shimadzu Prominence Modular HPLC system (Shimadzu Corporation, Kyoto, Japan) on a Kinetex^®^ 2.6mm C18 100 Å LC column (30 × 3 mm) at 45 °C. Mobile phase flow rate was 1.1 mL/min using a gradient of AcN and 0.1% (*v*/*v*) formic acid in water (from 5% AcN to 95% in 2.5 min). Chromatograms were recorded at the wavelength of 200–500 nm, integration was carried out at the UV_max_ of each compound. The applied injection volume was 6 μL.

### 3.3. Determination of Experimental logP Values

Lipophilicity measurements of hydrocurcumins were performed by dissolving the compounds in the organic solvents (octanol and toluene) at 1mg/mL concentration and mixing them with water (1:5 and 1:10 ratios, each in five replicates). After 24 h the concentration of the compounds in the aqueous phase were calculated by using a calibration dilution series measured on RP-HPLC system. The concentration of the organic phase was calculated from the concentration of the aqueous phase.

### 3.4. Antioxidant Activity

#### 3.4.1. Diphenyl-2-picrylhydrazyl (DPPH)-Assay

The DPPH free radical scavenging assay was performed based on the method of Fukumoto et al. [48] with some modification. The measurement was performed on a 96-well microplate. Microdilution series of the samples (0.2 mg/mL stock solution, dissolved in HPLC grade MeOH) were made starting with 100 μL. To each well 100 μL of DPPH reagent (100 μM in HPLC grade MeOH) was added to gain the final volume of 200 μL. The microplates were kept in dark, and the absorbance was measured after 30 min at 550 nm using a FluoStar Optima plate reader (software version 2.20R2, BMG Labtech, Ortenberg, Germany). Methanol was used as blank. The scavenging activity was calculated as Inhibition (%) = (A0 − As)/A0 × 100, and EC_50_ values were calculated by Graph Pad Prism (GraphPad Software, Inc., La Jolla, CA, USA).

#### 3.4.2. AAPH Assay

Antioxidant capacity measurement was carried out on 96-well microplates, based on oxygen radical absorbance capacity (ORAC) assay [49]. A 20 μL aliquot of the sample (6 dilutions with buffer from 10 μg/mL stock solution dissolved in phosphate buffer, containing 1% MeOH), 120 μL of fluorescein (dissolved in phosphate buffer, pH = 7.4, 70 nM final concentration), and 60 μL of 2,2′-azobis(2-methylpropionamidine) dihydrochloride (AAPH, dissolved in phosphate buffer, pH = 7.4, 12 mM final concentration) were pipetted into each well. Each measurement was performed twice in duplicates. Each plate contained six dilutions of trolox as the standard, and phosphate buffer as blank. Flourescence (excitation at 485 nm and emission at 520 nm) was measured over a 3 h period with 1.5-min cycle intervals by a FluoStar Optima plate-reader (BMG Labtech GmbH). The antioxidant capacity was calculated for each concentration with the following formula: antioxidant capacity = (AUC_Antioxidant_ − AUC_Blank_)/AUC_Blank_ × 100 where AUC is the area under curve obtained for the sample and blank assays. Based on the linear correlation between the concentration of the samples and the antioxidant capacity, IC_50_ values could be obtained, which define the concentration (in µM) of each compound required to have 50% antioxidant capacity defined by the previous equation.

## 4. Conclusions

Based on our results, hydrogenated curcumins could potentially be used as novel pharmacotherapeutics. They show better pharmacokinetic properties and higher bioavailability in various relevant models than curcumin. Among the tested compounds, hexahydrocurcumin was found by far the best lead for a possible further development: it had the best kinetic solubility, a good metabolic stability, appropriate GI and BBB penetration properties, and, importantly, the best lipophilic-ligand efficiency as an antioxidant.

Tetrahydrocurcumin and tetrahydrodemethoxycurcumin, however, demonstrated surprisingly low metabolic stability in human liver microsomes, and this was much lower than that of curcumin. Therefore, despite their otherwise good performance concerning some important physicochemical parameters (e.g., showing the best GI and BBB penetration), the applicability of tetrahydrocurcuminoids as possible lead compounds would rely on an appropriate formulation bypassing the first-pass metabolism.

## Figures and Tables

**Table 1:** Reaction conditions of the continuous flow hydrogenation of curcumin, and yields achieved for tetra-, hexa-, and octahydrocurcumin (DMC, 4HC, 6HC and 8HC respectively). p: pressure set on the instrument, EtOAc: ethyl acetate, T: temperature, STY: space time yield.

c (mg/mL)	Solvent	p	T	Flow Rate	Yields (%) ^#^			STY (mol/L/h) ^#^		
		(bar)	(°C)	(ml/min)	4HC	6HC	8HC	4HC	6HC	8HC
2	MeOH	10	RT	1	36.7	12.5	11.7	0.53	0.18	0.17
2	MeOH	40	RT	1	44.1	10.7	8.5	0.64	0.15	0.12
2	MeOH	80	RT	1	51.9	9.4	6.2	0.75	0.14	0.089
0.25	Toluene	0	RT	1	37.6	7.9	0.0	0.068	0.014	n.a.
0.25	Toluene	80	RT	1	38.2	14.2	1.2	0.069	0.026	0.002
0.25	Toluene	80	80	1	42.8	18.0	2.2	0.077	0.032	0.004
2	EtOAc	0	25	0.5	56.4	12.1	7.6	0.41	0.087	0.055
15	EtOAc:EtOH (1:1, *v*/*v*)	10	60	1	58.4	11.0	2.8	6.31	1.19	0.30

^#^ Determined by HPLC on the crude product mixtures by single-point experiments.

**Table 2:** Experimental physicochemical and in vitro pharmacokinetic profile of curcumin, demethoxycurcumin and their hydrogenated analogues. P_e_: effective permeability, MR: membrane retention. Kinetic solubility, in vitro human microsomal t_1/2_ and P_e_, and the MR values of permeability measurements represent mean ± S.E.M; *: *p* < 0.001 by one-way ANOVA and Bonferroni post-hoc test as compared to the corresponding parent compound, i.e., curcumin for 4HC, 6HC and 8HC, and DMC for 4HDC and 8HDC, *n* = 3.

Compound	Kinetic Solubility ^a^	Chemical Stability ^b^	Met.stab. in HLM ^c^ t_1/2/_Cl’_int_	BBB-PAMPA (PBS pH 6.5→pH 7.4)		GI-PAMPA (PBS pH 7.4)	
	(μM)	Shelf-Life (min)	(min)/(mL/min/kg)	Pe (·10^−7^ cm/s)	MR (%)	Pe (·10^−7^ cm/s)	MR (%)
Curcumin	3.1 ± 0.1	29.7 ± 0.6	70.5 ± 5.1/4.5 ± 0.3	NM ^d^	96.7 ± 0.4	5.0 ± 0.4	90.6 ± 0.3
DMC	5.7 ± 0.9	22.1 ± 0.2	37.8 ± 0.9/8.3 ± 0.2	NM ^d^	98.3 ± 0.1	10.6 ± 0.6	85.6 ± 0.5
4HC	70.3 ± 0.2 *	Stable ^#^	4.34 ± 0.01/71.8 ± 0.1 *	31.3 ± 1.1	34.4 ± 0.6 *	30.3 ± 1.3 *	13.3 ± 0.4 *
4HDC	69.5 ± 3.1 *	Stable ^#^	4.33 ± 0.02/72.0 ± 0.4 *	20.7 ± 0.5	40.9 ± 1.5 *	20.4 ± 0.3 *	12.9 ± 1.6 *
6HC	74.1 ± 0.3 *	Stable ^#^	94.4 ± 3,9/3.3 ± 0.2	12.5 ± 0.1	13.3 ± 0.5 *	3.7 ± 0.2 *	5.0 ± 0.6 *
8HC	72.9 ± 0.9 *	Stable ^#^	231.7 ± 33.6/1.4 ± 0.2 *	9.7 ± 0.2	8.9 ± 1.4 *	2.4 ± 0.1 *	4.4 ± 0.2 *
8HDC	68.7 ± 0.7 *	Stable ^#^	365.1 ± 57.4/0.9 ± 0.2 *	5.3 ± 0.7	14.9 ± 1.9 *	2.2 ± 0.1 *	1.7 ± 0.3 *

^a^ after 2 h, at 37 °C in PBS, pH 7.4; ^b^ between 0–2 h at 37 °C in PBS, pH 7.4; ^c^ Metabolic stability in human liver microsomes (HLM); intrinsic clearance (Cl’_int_) values were calculated from in vitro t_1/2_ data in HLM system as described in the Materials and Methods section. ^d^ The permeability value could not be measured due to the high membrane retention. ^#^ No chemical change was detected during the evaluation time (4 h).

**Table 3:** Predicted physicochemical and ADME properties of curcumin and hydrocurcumin analogues (using ACD/Labs Percepta software [42]). P_app_: apparent permeability. HBD: number of hydrogen bond donors. CNS MPO: Central Nervous System Multiparameter Optimization introduced by Wager et al. [43].

Compound	Aqueous Solubility ^a^ (mg/mL)	logP/logD_7.4_^b^	TPSA (Å^2^)	HBD	Strongest *pK_a,acid_* ^b^	CNS MPO ^c^	logBB ^b^	Caco-2 Permeability P_app_ (10^−6^ cm/s)
Curcumin	0.08	2.64/2.58	96.2	3	8.3	4.66	0.03	93
DMC	0.07	2.85/2.79	87.0	3	8.3	4.91	0.18	99
4HC	0.10	2.41/2.38	96.2	3	8.7	4.49	0.01	45
4HDC	0.15	2.79/2.76	87.0	3	8.7	4.66	0.21	54
6HC	0.92	1.76/1.76	96.2	3	10.0	4.67	−0.21	28
8HC	0.22	2.18/2.18	99.4	4	9.8	4.21	−0.18	17
8HDC	0.30	2.48/2.48	90.2	4	10.1	4.52	−0.25	22

^a^ intrinsic solubility using Drug Profiler unit of Percepta Package. ^b^ Calculated using logP (Consensus and p*K_a_* (Classic) settings within Percepta package ^c^ CNS MPO values were determined using predicted logP, logD_7.4_, TPSA and HBD values.

**Table 4:** Lipophilicity of curcumin and hydrocurcumin analogs in two different biphasic (octanol—water: logP_oct/w_ and toluene—water: logP_tol/w_) systems. Data represent average ± S.E.M from five parallel experiments.

Compound	logP_oct/w_	logP_tol/w_	ΔlogP_oct_tol_
4HC	2.58 ± 0.07	2.27 ± 0.05	0.31
6HC	2.15 ± 0.01	1.69 ± 0.03	0.46
8HC	2.30 ± 0.02	1.55 ± 0.14	0.75

**Table 5:** Antioxidant activity of hydrocurcumin derivatives. Data represent average ± S.E.M; *: *p* < 0.05, ***: *p* < 0.001 by one-way ANOVA and Bonferroni post-hoc test as compared to the corresponding parent compound, i.e., curcumin for 4HC, 6HC and 8HC, and DMC for 4HDC and 8HDC; ^##^: *p* < 0.01, ^###^: *p* < 0.001 by one-way ANOVA and Bonferroni post-hoc test as compared to trolox, *n* = 6–10.

Compound	DPPH IC_50_ µM	LLE ^a^ (DPPH)	Antioxidant capacity (AAPH) IC_50_ µM	LLE (AAPH)
Curcumin	11.8 ± 0.1	2.37 ± 0.004 ^###^	0.32 ± 0.10 ^###^	3.96 ± 0.05 ^###^
DMC	16.8 ± 0.7	2.09 ± 0.04 ^###^	0.49 ± 0.03 ^###^	3.63 ± 0.03 ^##^
4HC	9.8 ± 0.2	2.60 ± 0.01	0.43 ± 0.04 ^###^	3.99 ± 0.05 ^##^
4HDC	14.2 ± 0.5	2.06 ± 0.02 ^###^	0.41 ± 0.03 ^###^	3.60 ± 0.04 ^##^
6HC	9.9 ± 0.9	3.25 ± 0.04 ***^, ###^	0.39 ± 0.06 ^###^	4.70 ± 0.08 ***^, ###^
8HC	11.4 ± 1.0	2.76 ± 0.04 ***	0.43 ± 0.11 ^###^	4.28 ± 0.11 *^, ##^
8HDC	33.1 ± 0.8 ^###^	2.00 ± 0.07 ^###^	0.49 ± 0.29 ^###^	3.64 ± 0.12 ^###^
Trolox	5.6 ± 0.1	2.74 ± 0.01	2.02 ± 0.13	3.18 ± 0.03

^a^ Ligand-lipophilic efficiency: LLE = pIC_50_—logP_predicted_

## Data Availability

Raw datasets are available from the authors upon request.

## Supplementary Materials

The following are available online. Table S1. LC-MS metabolite fingerprint data of each compound following their incubation with human liver microsomes, Figures S1—S7. ^1^H NMR spectra of the compounds.

## Sample Availability

Samples of each compound evaluated in this study are available from the authors.
