# Supplementary material for: Pharmacokinetics-Driven Evaluation of the Antioxidant Activity of Curcuminoids and Their Major Reduced Metabolites—A Medicinal Chemistry Approach
Source: Molecules. 2021 Jun 10;26(12):3542. doi: 10.3390/molecules26123542 (PMC8229286; doi:10.3390/molecules26123542)
Supplement: Supplementary file 1 [file molecules-26-03542-s001.zip › molecules-1225369-supplementary.pdf]

Supporting Information

Pharmacokinetics-driven evaluation of the antioxidant activity of curcuminoids and their major reduced metabolites – a medicinal chemistry approach

Gábor Girst[1], Sándor B. Ötvös[2,3], Ferenc Fülöp[2], György T. Balogh[4,5,*], Attila Hunyadi[1,6,*]

[1] Institute of Pharmacognosy, Interdisciplinary Centre of Excellence, University of Szeged, H-6720 Szeged, Hungary; girst.gabor@pharmacognosy.hu (G.G.)
[2] Institute of Pharmaceutical Chemistry, University of Szeged, H-6720 Szeged, Hungary; (sandor.oetvoes@uni-graz.at) (S.B.Ö.); fulop.ferenc@szte.hu (F.F.)
[3] Institute of Chemistry, University of Graz, NAWI Graz, Heinrichstrasse 28, A-8010 Graz, Austria.
[4] Department of Chemical and Environmental Process Engineering, Budapest University of Technology and Economics, H-1111, Budapest, Hungary
[5] Department of Pharmacodynamics and Biopharmacy, University of Szeged, H-6720, Szeged, Hungary
[6] Interdisciplinary Centre of Natural Products, University of Szeged, H-6720 Szeged, Hungary
* Correspondence: hunyadi.a@pharmacognosy.hu (A.H.); balogh.gyorgy@vbk.bme.hu (G.T.B.).; Tel.: +3662546456 (A.H.); +3614632174 (G.T.B.)

Table of Contents:

Table S1. LC-MS metabolite fingerprint data of each compound following their incubation with human liver microsomes. 2
Figure S1. ^{1}H NMR spectrum of curcumin studied in this work........3
Figure S2. ^{1}H NMR spectrum of demethoxycurcumin (DMC) studied in this work........4
Figure S3. ^{1}H NMR spectrum of tetrahydrocurcumin (4HC) studied in this work........5
Figure S4. ^{1}H NMR spectrum of tetrahydro-demethoxycurcumin (4HDC) studied in this work........6
Figure S5. ^{1}H NMR spectrum of hexahydrocurcumin (6HC) studied in this work........7
Figure S6. ^{1}H NMR spectrum of octahydrocurcumin (8HC) studied in this work........8
Figure S7. ^{1}H NMR spectrum of octahydro-demethoxycurcumin (8HDC) studied in this work........9

Table S1. LC-MS metabolite fingerprint data of each compound following their incubation with human liver microsomes. Retention time (tR), characteristic m/z values observed in the mass spectrum, and relationship of the base peak with the parent compound's molecular ion m/z are presented.

tR	Curcumin m/z (369.3)	DeMeOcurcumin m/z (339.2)	4HC m/z (373.3)	4HDC m/z (343.2)	6HC m/z (375.2)	8HC m/z (377.3)	8HDC m/z (346.2)
1.73	**401.2**/256.2 = M + 32						
1.96							**385.2**#?/247.2 = M + 16?
2.02					**355.2**/373.1 = M-2?		
2.03							**367.2**# = M - 2
2.11						**401.2**#/361.3/343.2/329.1 = M + 2	
2.13				**369.3**#/311.3 = M + 4 (8HDC)			**369.2**#/311.2 = M
2.14			**355.2**/373.2 = M isomer?		**355.1**/373.1/395.3# = M-2		
2.17			**341.3**/399.3# = M + 4		**341.3**/399.2# = M+4	**341.2** = M isomer?	**325.3**
2.18		**327.2**/176.0 = M - 12?					
2.19					**341.3**/399.2# = M+2 (8HC)	**399.3**#/341.2 = M	
2.21		**325.1**/256.3 = M - 14?		**367.3**# /327.3/177.2 = M+2			
2.22							**375.2**#/325.3 = M + 6?
2.23	**355.3**/256.3 = M - 14						
2.25	**[illegible]57.2**/397.2/256.0 = M + 6 (6HC)		**357.2**/397.2# = M+2 (6HC[illegible]	**[illegible]7.2**/397.2#/325.3 = 4HC + 2 (6H[illegible]	**397.3**#/357.2/325.2/177.2 = M	**357.3** = M-2 (6HC)	
2.27		**343.2**/365.2# = M + 4					
2.30	**[illegible]73.3**/395.3#/340.2/256.2 = M+4						
2.35				**325.3**/343.2/365.2# = M			
2.40			**355.3**/395.2#/177.2 = M	**325.1**/355.2 = M+30 (4HC)			
2.76			**371.3** = M-2				
2.81				**325.3**/343.2/365.2# = M			
2.84			**355.3**/395.2# = M	**325,3**/355.3 = M+30 (4HC)			
2.92		**341.2**/256.3 = M+2					
2.95	**371.2**/256.2 = M+2						
3.00		**339.2**/256.3 = M					
3.05	**369.3***/256.2/391.2#/177.0 = M						
3.08				**288.4**			
3.13			**256.4**/774.4				

Mother compound: M (green)

Major metabolites based on the peak area detected at 220 +/- 4 nm (orange)

Bold: Base (or most intensive) peak/ ion with the greatest relative abundance

*: m+H

#: m+Na

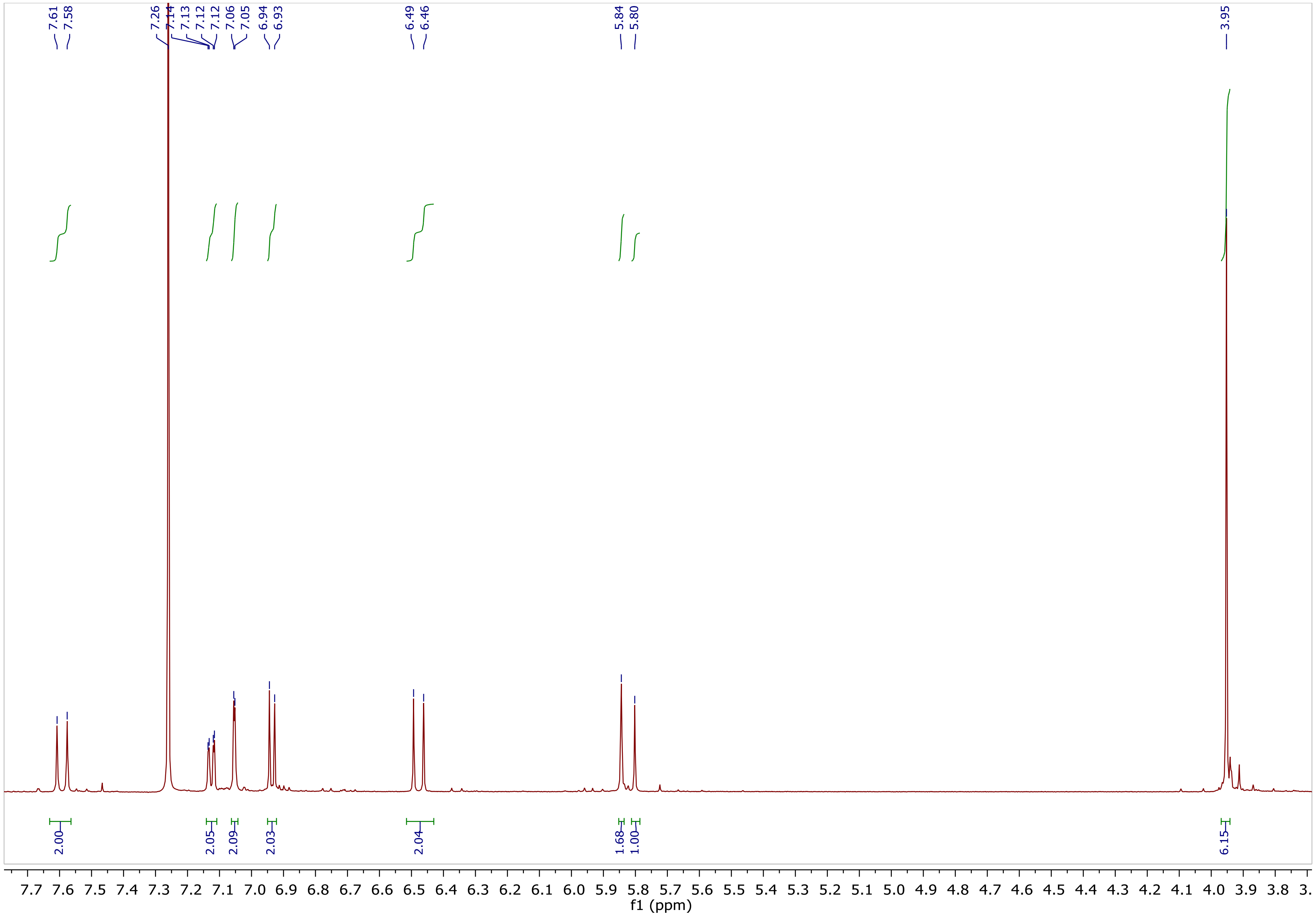

Figure S1. ^{1}H NMR spectrum of curcumin studied in this work (500MHz, CDCl3).

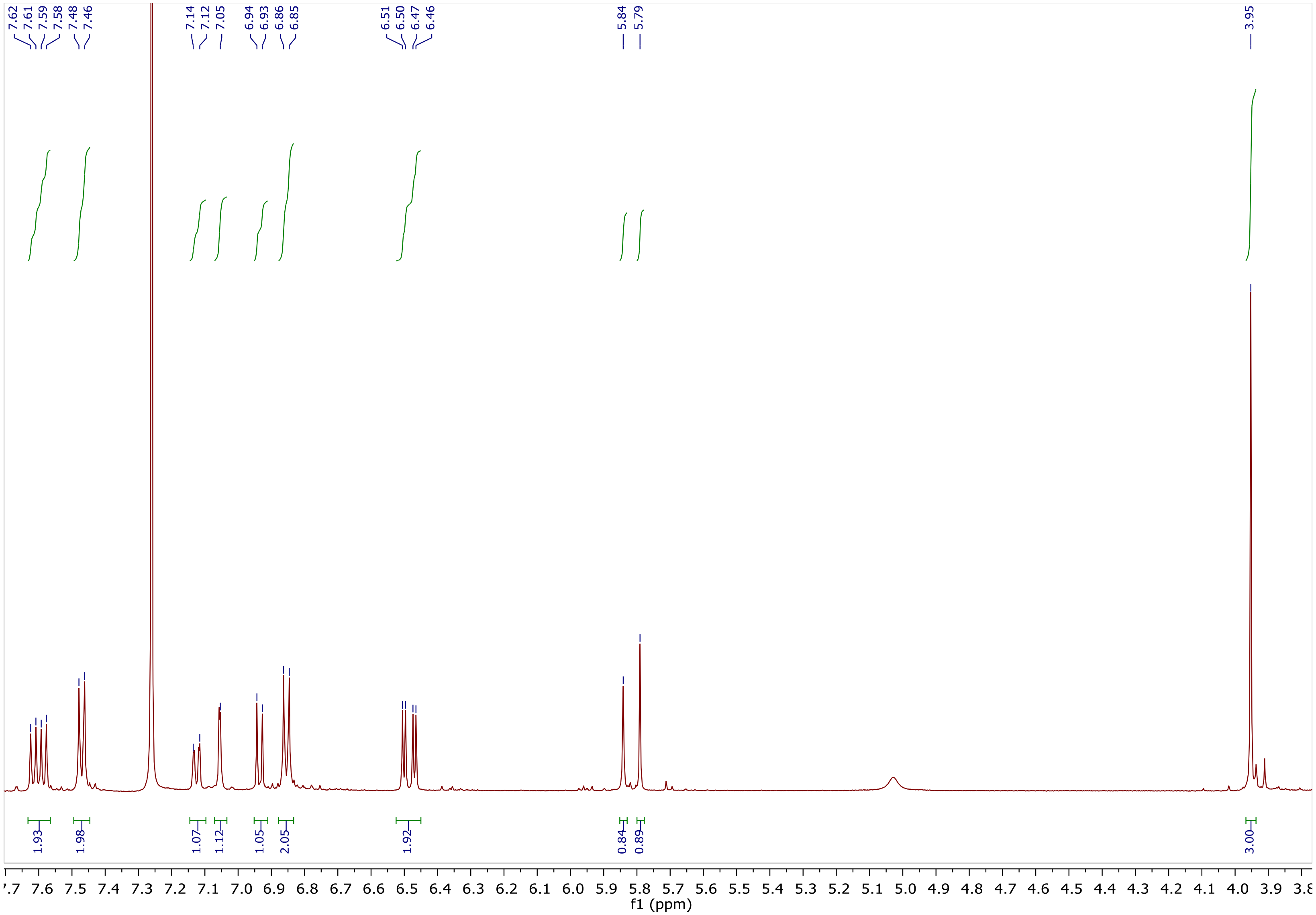

Figure S2. ^{1}H NMR spectrum of demethoxycurcumin (DMC) studied in this work (500MHz, $CDCl_3$).

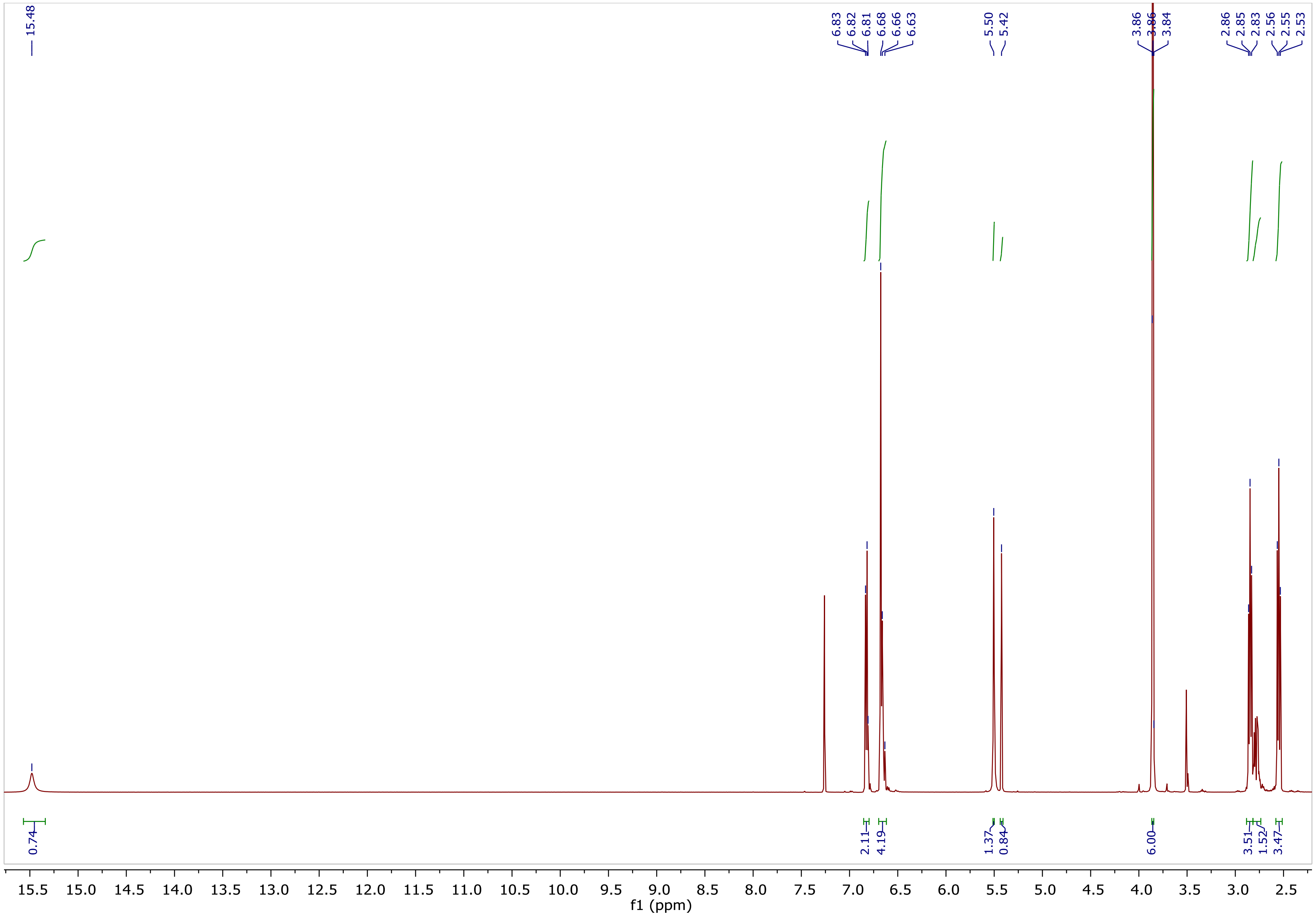

Figure S3. ^{1}H NMR spectrum of tetrahydrocurcumin (4HC) studied in this work (500MHz, $CDCl_3$).

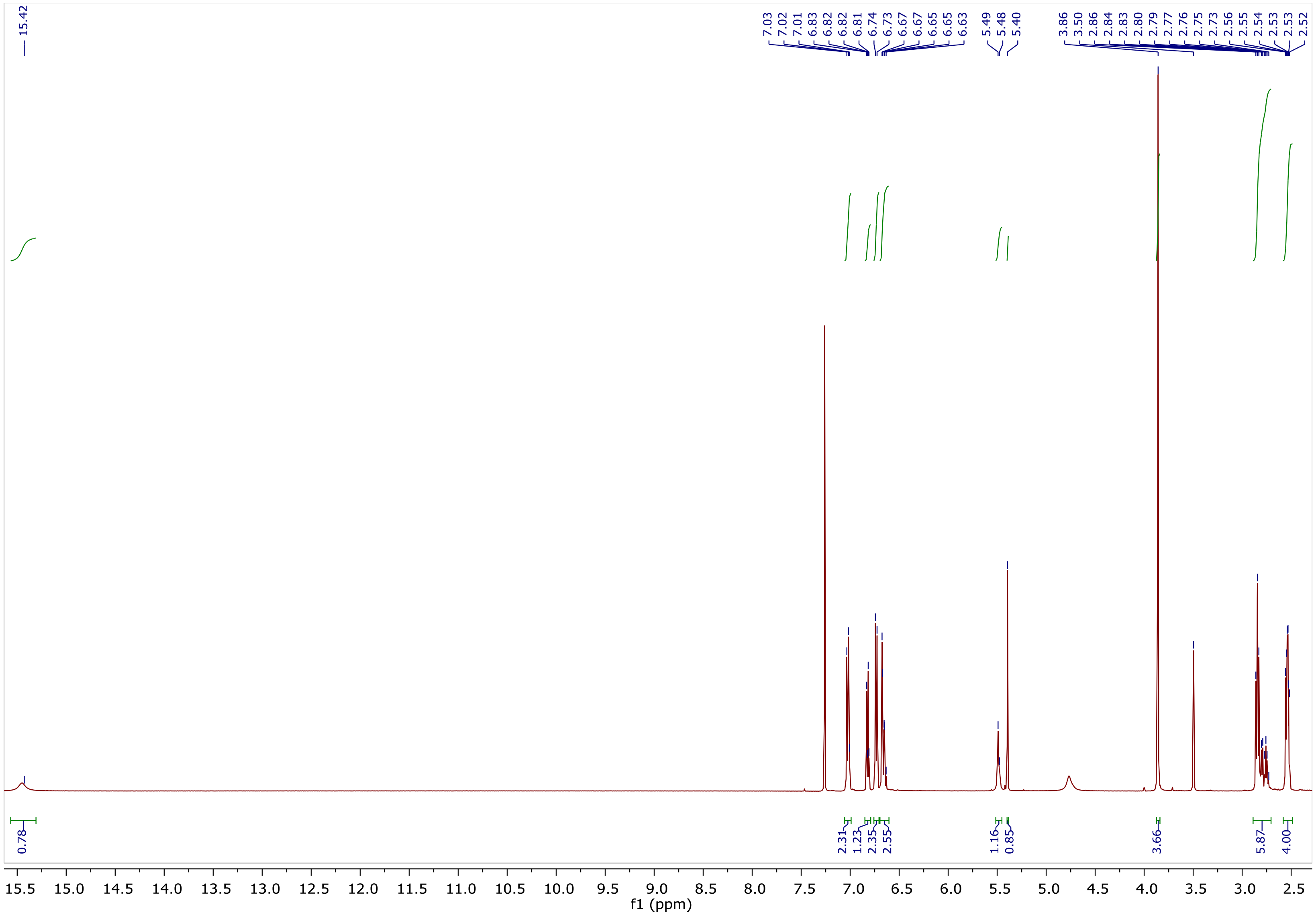

Figure S4. ^{1}H NMR spectrum of tetrahydro-demethoxycurcumin (4HDC) studied in this work (500MHz, $CDCl_3$).

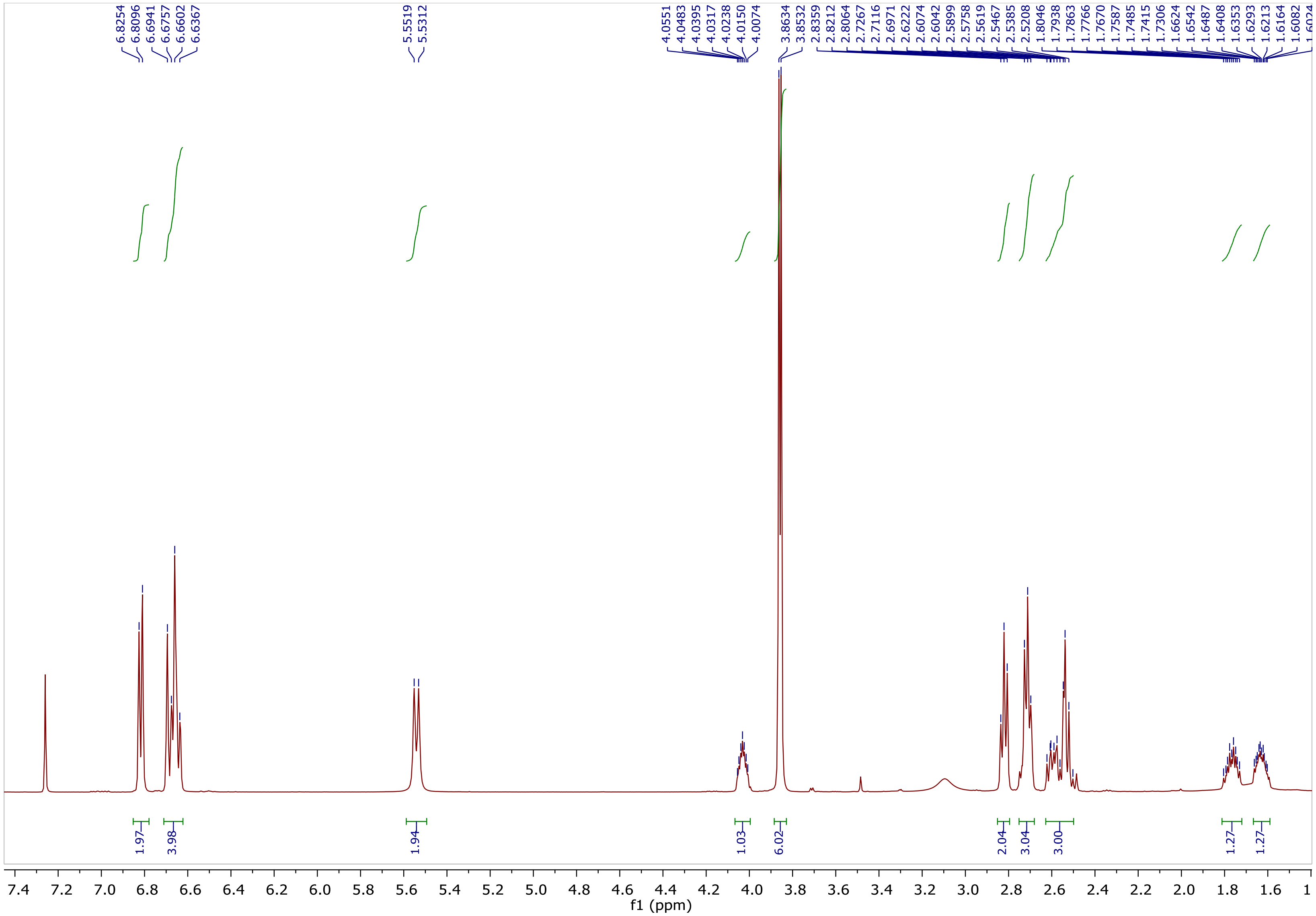

Figure S5. ^{1}H NMR spectrum of hexahydrocurcumin (6HC) studied in this work (500MHz, $CDCl_3$).

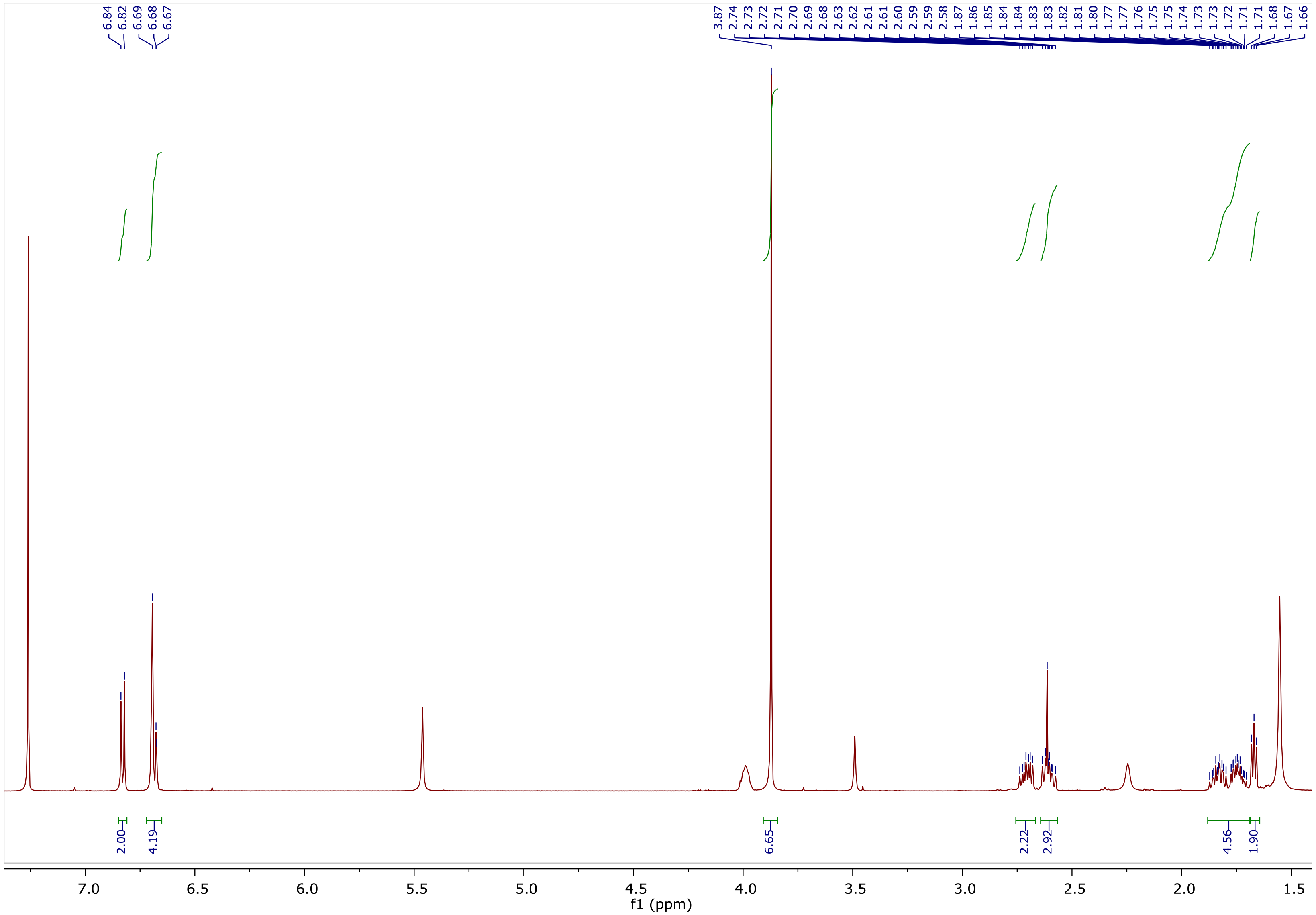

Figure S6. ^{1}H NMR spectrum of octahydrocurcumin (8HC) studied in this work (500MHz, $CDCl_3$).

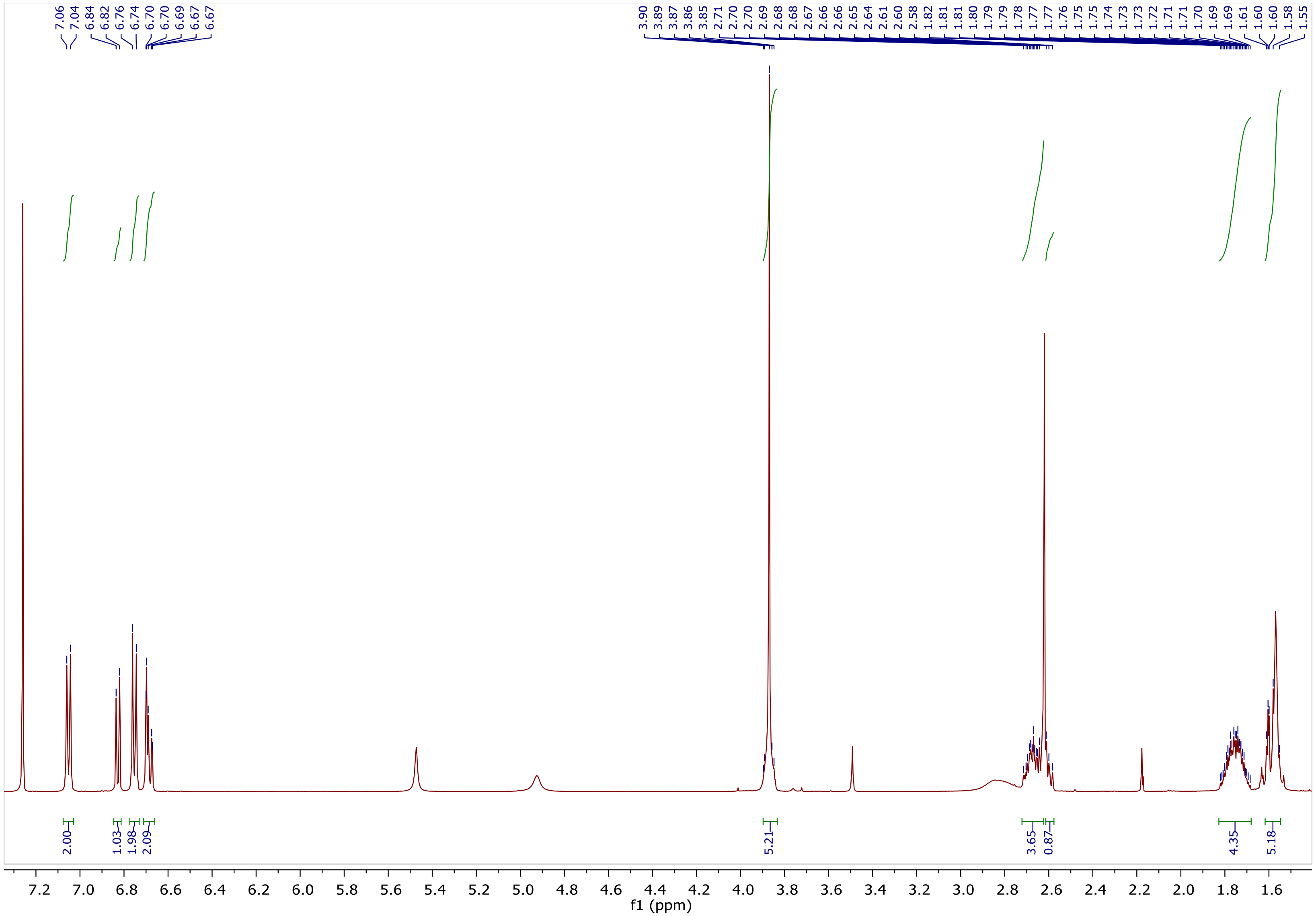

Figure S7. ^{1}H NMR spectrum of octahydro-demethoxycurcumin (8HDC) studied in this work (500MHz, $CDCl_3$).
